# Immune and Metabolic Mechanisms of Endothelial Dysfunction

**DOI:** 10.3390/ijms252413337

**Published:** 2024-12-12

**Authors:** Irakli Kopaliani, Basant Elsaid, Stephan Speier, Andreas Deussen

**Affiliations:** 1 Institute of Physiology, Medical Faculty Carl Gustav Carus, Technische Universität Dresden, 01037 Dresden, Germany; basantshaker@med.asu.edu.eg (B.E.); stephan.speier@tu-dresden.de (S.S.); andreas.deussen@tu-dresden.de (A.D.); 2 Department of Physiology, Faculty of Medicine, Ain Shams University, Cairo 1181, Egypt; 3 Paul Langerhans Institute Dresden of the Helmholtz Zentrum München at the University Clinic Carl Gustav Carus of Technische Universität Dresden, Helmholtz Zentrum München, 85764 Neuherberg, Germany

**Keywords:** endothelium, inflammation, metabolism, endothelial dysfunction, vessel remodelling, hypertension, sex-differences

## Abstract

Endothelial dysfunction is a strong prognostic factor in predicting the development of cardiovascular diseases. Dysfunctional endothelium loses its homeostatic ability to regulate vascular tone and prevent overactivation of inflammation, leading to vascular dysfunction. These functions are critical for vascular homeostasis and arterial pressure control, the disruption of which may lead to hypertension. Hypertension itself can also cause endothelial dysfunction, as endothelial cells are susceptible to haemodynamic changes. Although it is unclear which of those factors appear first, they create a vicious circle further damaging multiple organs, including the heart and vessels. There are also sex-specific differences in homeostatic functions of the endothelium regarding vessel tone regulation, which may contribute to differences in arterial blood pressure between men and women. Even more importantly, there are sex-differences in the development of endothelial dysfunction and vessel remodelling. Hence, an understanding of the mechanisms of endothelial dysfunction and its contribution to pathological vascular remodelling during hypertension is of critical importance. This review addresses immunological and metabolic aspects in mechanisms of endothelial dysfunction and the resulting mechanisms in vascular remodelling with respect to arterial hypertension, including the potential role of sex-specific differences.

## 1. Introduction

Endothelial and vascular dysfunctions are hallmarks of hypertension. While it is still unresolved whether endothelial dysfunction leads to development of hypertension or endothelial dysfunction is merely a consequence of hypertension, inflammation and metabolic stress are the driving forces of endothelial dysfunction in hypertension. While endothelial cells are considered a part of the innate immune system, fine physiological changes in its function are essential for initiation and propagation of inflammation, as well as in ceasing the inflammatory process. Endothelial dysfunction alters this physiological interaction between immune and endothelial cells, leading to an imbalance where dysfunctional endothelial cells aggravate inflammatory recruitment into the vessel wall and inflammatory cells with their cytokines worsen endothelial function. Because hypertension is often accompanied by metabolic diseases, the separation of the extent of metabolic and inflammatory burden on endothelial cell function is not an easy task. Furthermore, the immune system plays a major role in the mediation of cellular and organ dysfunction irrespective of whether hypertension is manifested with or without metabolic diseases. Nevertheless, from the therapeutic perspective, it is critical to understand how altered immune and metabolic states affect homeostatic functions of endothelial cells and cause disease progression.

### 1.1. Basic Structure of the Endothelium

**Figure 1:**
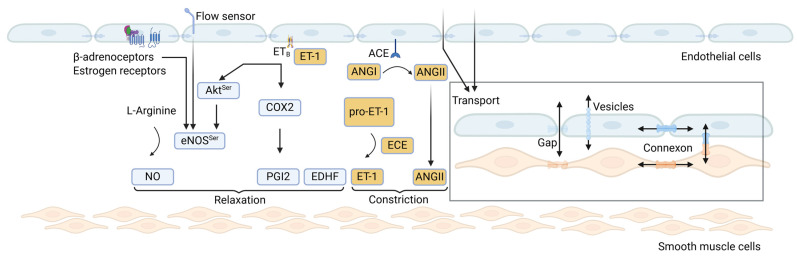
Vasoactive factors produced by endothelial cells and the contribution of the endothelium in the conversion of ANGI to ANGII and substrate transport related to the endothelium. Created with Biorender (Toronto, Canada).

The endothelium, which lines the entire vasculature in all tissues and organs, is made up of a single layer of cells with an average size of 20–40 µm in length, 10–15 µm in width, and 0.1–0.5 µm thickness. Originally considered merely a layer of semipermeable membrane, today the endothelium is estimated to have a total mass of approximately 1.5 kg as the largest endocrine organ in human body, whose normal functioning is critical for vascular health [1,2,3]. Conversely, a dysfunctional endothelium is a crucial trigger of vascular disease. Although the endothelium appears as a homogeneous monolayer of the vessel wall and shares a molecular signature across the vasculature, organ specific endothelial heterogeneity and plasticity is evident. This heterogeneity defines differences in major endothelial functions such as permeability, response to various components of the circulating blood or tissue microenvironments, and synthesis of various factors [1,2]. With respect to permeability, the endothelium controls transport of molecules across the capillary wall by different mechanisms. Macromolecules can diffuse within the endothelial cell layer via connexons, which create channels for intercellular and transcellular transport (Figure 1). Extracellular diffusion occurs through endothelial gaps made by cell-to-cell junctions across the endothelial cell layer. In addition, vesicular transport is a mechanism for delivering macromolecules across the endothelium [4]. As the size of most biomolecules is large and diffusion coefficients are lower within endothelial cells, diffusion through intercellular junctions is thought to be a major transport mechanism across the endothelium, excluding the brain vascular endothelium, which makes up for an important part of the blood–brain barrier.

### 1.2. Basic Functions of the Endothelium

#### 1.2.1. Effects on Vessel Tone and Permeability

For the regulation of local vascular tone, endothelial–smooth muscle cell crosstalk plays an important physiological role. There are several vessel relaxing factors such as NO, prostaglandin (PGI2) and endothelium-derived hyperpolarising factors (EDHFs) produced by endothelial cells (Figure 1) [5,6]. eNOS, a heme-containing enzyme is activated by calcium–calmodulin and synthesises NO from L-arginine. Despite its very short half-life, endothelium-released NO effectively acts on smooth muscle cells where it activates the cGMP/PKG pathway causing Ca^2+^ sequestration and smooth-muscle cell relaxation with consequent vasodilatation [7]. The sympathetic neurotransmitter norepinephrine is a major regulator of vessel tone in the entire vascular tree. Smooth muscle cell-located α- and β-adrenoceptors are well-known targets for neuronal norepinephrine and humoral epinephrine causing vasoconstriction and vasorelaxation, respectively. However, besides smooth muscle cells, α- and β-adrenoceptors are also present on endothelial cells [8,9,10]. Upon stimulation with norepinephrine, they exert endothelium-dependent NO-mediated effects on vessel tone [11,12,13,14]. This makes the endothelium a target and a constituent of sympathetic vessel tone regulation.

The endothelium is well known as a source of NO, which has antioxidant, anti-proliferative, anti-inflammatory, and platelet inhibitory effects [7,15]. Thereby, NO supports vascular homeostasis. The most probable relevant stimulus in the regulation of eNOS activity is shear stress. In accordance with this, shear stress responsive elements are found in the eNOS promoter region on DNA [16]. eNOS catalytic activity is regulated by agonists such as bradykinin and thrombin via their respective receptors [17]. Underlying mechanisms of eNOS activation include changes in endothelial cell Ca^2+^-concentration or phosphorylation of the eNOS protein (Figure 1).

Sex hormones are critical regulators of endothelial NO production. Both oestrogen and testosterone seem to increase eNOS activity and consequently NO bioavailability [18]. Interestingly, testosterone actions are partly mediated after its aromatase-dependent conversion to 17β-oestradiol [19]. It is worth noting, however, that 6β-hydroxytestosterone, a metabolite of testosterone, has been reported to mediate endothelial dysfunction and vessel remodelling in a model of ANGII-induced hypertension [20]. Other hormones, such as insulin and growth hormone, increase NO bioavailability, whereas progesterone and glucocorticoids decrease it [18].

In many experimental setups, the combined inhibition of NO and PGI2 does not completely block vessel relaxation upon pharmacological stimulation, e.g., with acetylcholine [5]. The remaining relaxation is attributed to the action of less well-defined EDHF(s). Functionally, EDHF(s) open K^+^ channels on smooth muscle cells and increase K^+^ conductance/efflux, resulting in smooth muscle cell membrane hyperpolarisation. There are several factors considered to act as EDHF(s), K^+^ itself, c-type natriuretic peptide, and cytochrome P450 metabolites. There is also electrical coupling through myoendothelial gap junctions, which appears more relevant in small resistant vessels and less in large conduit arteries [21,22,23]. Besides their physiological action on vessel tone, EDHF(s) appear especially important in compensating for decreases in NO bioavailability. Along this line, despite of a decrease in NO bioavailability, endothelium-dependent dilatation of the radial artery is maintained in patients with hypertension, which is attributed to a compensatory action of EDHF(s) [22,24]. A similar compensatory effect is observed in patients with hypercholesterolemia, where NO-mediated vessel relaxation is decreased, whereas EDHF(s)-mediated relaxation is preserved [25]. Atherosclerotic damage of the vessel wall, however, seems to result in a loss of EDHF(s)-mediated vessel relaxation, pointing to the potential importance of the extent or mode of endothelial damage as critical factors in loss of its function [25].

Besides vasodilators such as NO, PGI2, and EDHFs, the endothelium produces endothelin-1 (ET-1), the most relevant form of ETs in vasculature, which is cleaved from its inactive pro-endothelin via endothelin converting enzyme (ECE) [26]. ET-1 is a very potent vasoconstrictor mediating its effects via ET_A_ and ET_B_ receptors, which are both expressed on vascular smooth muscle cells. Interestingly, ET_B_ receptors are also expressed on endothelial cells, which, upon activation, stimulate endothelial PGI2 and NO release [26,27]. Under physiological conditions, ET-1 and NO mediate opposing effects in terms of vessel tone regulation. In addition, they are interdependent. NO ameliorates ET-1 production, whereas ET-1 is a strong antagonist to NO-dependent vasodilatation. Under baseline physiological conditions, endothelial ET-1 formation is low. However, in endothelial dysfunction, ET-1 production is enhanced, while NO availability is reduced. In addition to its immediate vasoconstrictive effect, ET-1 may also promote vascular smooth muscle cell hypertrophy and proliferation and exert pro-inflammatory actions in vessels [28,29]. Hence, pharmacological ET_A_ and ET_B_ receptor blockade is used in the management of vascular disease associated with vessel wall remodelling [30].

#### 1.2.2. Constitutive Role in Arterial Pressure Control

In addition to vasoconstrictive ET-1, the endothelium regulates the formation of angiotensin II (ANGII), by expressing angiotensin converting enzyme (ACE), which converts inactive ANGI into effector ANGII. Angiotensin II (ANGII) is another strong vasoconstrictor that belongs to the arterial pressure controlling renin–angiotensin system (RAS). Although ANGII is not produced by endothelial cells, they express the critical enzyme ACE, which converts inactive ANGI to effector ANGII [31]. Besides systemic RAS, which produces circulating ANGII, local RAS is present in tissues. All components of RAS, except renin, are produced in the vasculature (Figure 1). Therefore, endothelial ACE regulates production of ANGII both systemically in the circulating blood and locally in the tissue. ANGII exerts its effects via ANGII type 1 (AT_1_R) and type 2 (AT_2_R) receptors. Both receptors are expressed in endothelial cells, making them not only a critical contributor to systemic and local formation of ANGII, but also a target of this factor [32,33]. ANGII typically derails protective and vasodilatory functions of endothelium, generally by supressing NO production, enhancing formation of reactive oxygen species (ROS) [34], and expressing inflammatory adhesion molecules, which favour vascular inflammation [35]. Finally, the endothelium also produces vasoconstrictive prostaglandins, e.g., PGF2α.

## 2. Mechanisms of Endothelial Dysfunction

### 2.1. ANGII-Induced Hypertension and Endothelial Dysfunction

ANGII, the key effector of RAS, has multiple actions on the cellular and organ levels. ANGII is a strong vasoconstrictor acting especially on small resistance vessels. Its constrictive action increases total peripheral resistance and thus arterial pressure. ANGII also induces the release of aldosterone, which in turn causes sodium and water retention. This ultimately increases intravascular volume, an important variable in long-term regulation of arterial pressure. Acting through its AT1R, ANGII exerts many other effects besides controlling arterial pressure. ANGII induces hypertrophy and hyperplasia of vascular smooth muscle cells, leading to vessel media hypertrophy. It also has hypertrophy stimulating actions on cardiomyocytes, leading to concentric cardiac hypertrophy [36]. ANGII increases basal ROS production and aggravates oxidative stress by PKC and PLA2 pathways, which are upstream regulators of NADPH oxidase [37,38,39].

**Figure 2:**
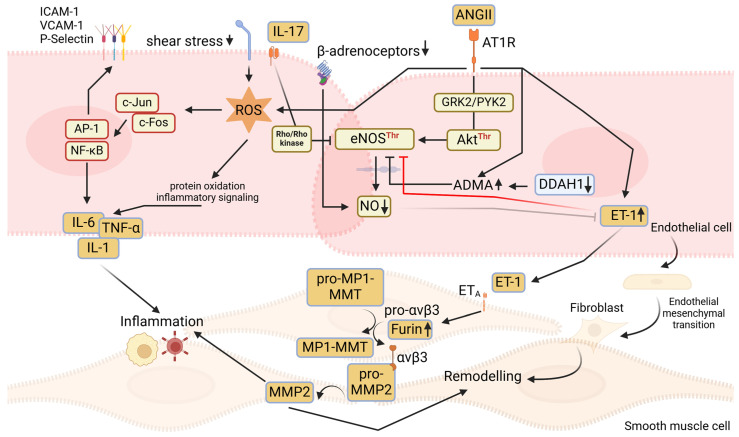
Mechanisms of endothelial dysfunction and contribution of dysfunctional endothelium to vessel remodelling.

Several studies demonstrate that ANGII promotes endothelial dysfunction by decreasing NO production [40,41,42,43,44]. Acting via its AT1R, ANGII increases eNOS phosphorylation at Thr and decreasing Ser phosphorylation (Figure 2). Increase in the Thr/Ser phosphorylation ratio inhibits eNOS activity and decreases NO production. The endothelial ANGII/AT1R axis acts via several downstream kinases. Taguchi et al. demonstrated that ANGII/AT1R activates GRK2 followed by Akt^Thr^ and eNOS^Thr^ phosphorylation and inhibition of NO production [42]. Loot et al. demonstrate activation of another kinase, PYK2, acting downstream of the ANGII/AT1R axis [40]. These two reports complement each other because PYK2 is upstream of Akt. Collectively, these studies demonstrate endothelial ANGII/AT1R/GRK2 and PYK2 as mechanistic links leading to adverse phosphorylation of eNOS and consequent inhibition of NO production. Because the endothelial Akt/eNOS axis is of importance for physiological production of NO, ANGII via AT1R and subsequent signalling pathways hijack this axis resulting in phosphorylation of eNOS on its inhibitory Thr residue, thereby decreasing NO production. Besides directly hijacking this physiological mechanism of NO production, ANGII also activates endothelial mechanisms supressing NO bioavailability. For example, Pang et al. demonstrate that ANGII induces nuclear translocation of Yes-associated protein, which binds to the transcription factor Tead1 and upregulates Galectin-3 (Gal-3) expression and supresses NO bioavailability [45].

Besides acting directly on endothelial AT1R and causing endothelial dysfunction, ANGII may act indirectly via asymmetric dimethyl arginine (ADMA), which is an endogenous eNOS inhibitor [46]. Increased ADMA levels inversely correlate with forearm blood flow and are associated with enhanced cardiovascular morbidity and mortality in patients with various cardiovascular risks [47,48]. ADMA levels also correlate with arterial remodelling as suggested by the association of ADMA levels with intima-medial thickness in patients with hypertension [49]. Thus, ADMA is accepted as representing an important marker of endothelial dysfunction as well as a prognostic marker for cardiovascular diseases. Dimethylaminohydrolase 1 (DDAH1) is an endogenous enzyme metabolising ADMA [50]. Lowering ADMA levels in genetic mouse models overexpressing DDAH1 protects from an ANGII-mediated increase in ADMA levels and cardiovascular remodelling [51,52]. These protective effects were associated with improved endothelial function in transgenic mice compared to WT controls after ANGII infusion. However, the protective effect of DDAH1 overexpression in endothelial function and cardiovascular remodelling was dependent on the dose and duration of ANGII infusion. After a high ANGII dose and long infusion period, transgenic mice did not show protection from endothelial dysfunction and cardiovascular remodelling. It is noted here that not all studies report increases in ADMA plasma levels in experimental hypertension models [51,52,53,54], which may at least partly reflect different protocols of the induction of hypertension. It may be of interest that DDAH1 may protect from endothelial dysfunction also via an ADMA independent pathway. Along this line, Sun et al. reported that DDAH1 phosphorylates Akt1^Ser^ leading to eNOS activation and NO production in vascular endothelial cells [55]. Furthermore, Liu et al. reported supportive findings on pulmonary endothelial cells where DDAH1 increased Akt^Ser^ phosphorylation and NO production [56]. Altogether, ANGII is a strong promoter of endothelial dysfunction acting via the AT1R causing inhibition of eNOS activity and NO production. The overwhelming evidence derived from experimental studies using ANGII-infusion in hypertension models with lower grade RAS activation demonstrates the association of endothelial dysfunction with cardiovascular remodelling [57].

Decreased NO production is not the only mechanism by which a dysfunctional endothelium contributes to vessel remodelling. Dysfunctional endothelium can differentiate into fibroblasts or smooth muscle cells and hence contribute to vessel fibrosis or hypertrophy. This transition of endothelial cells requires highly complex molecular and phenotypic changes. Some mechanisms have recently been reported. Pharmacological inhibition or genetic deletion of myeloid differentiation protein 2 (MD2) attenuates ANGII-induced endothelial to mesenchymal transition in a mouse model and protects from vascular dysfunction [58]. MD2 seems to act via endothelial NFkB, which, in turn, is a known pro-inflammatory transcription factor. Another study by Lin et al. also reported the mechanistic role of NFkB in endothelial mesenchymal transition [59]. This study, however, proposed a role of toll like receptor 2 (TLR2) and an ANGII/TLR2/NFkB mechanistic model in endothelial mesenchymal transition. Finally, Shengban et al. demonstrate that non-pharmacological inhibition of NFkB ameliorates ANGII-induced endothelial mesenchymal transition in mice and protects from vascular remodelling [60].

### 2.2. Inflammation and Oxidative Stress

#### 2.2.1. Reactive Oxygen Species

ROS are important by-products of cellular metabolism. They act as second messengers in cellular signalling and represent important protective factors in the anti-bacterial immune defence system. Hence, under homeostatic conditions, ROS play important physiological roles. However, if ROS production exceeds normal levels in various disease conditions, it may cause endothelial dysfunction and even promote apoptotic endothelial cell death [61,62]. ROS cause oxidative cell membrane damage by the process of lipid peroxidation which removes electrons from lipids and damages phospholipids. Increased ROS, especially the anion superoxide, result in the formation of peroxynitrite, thereby eliminating NO and promoting protein nitration.

Besides mitochondrial oxidative metabolism, major sources of ROS in the vasculature are various forms of NADPH oxidases, with the possible exception of NADPH4, which decreases ROS [63]. The expression of several isoforms of NADPH oxidase is increased during hypertension and the increase in ROS may cause dysregulation of endothelium-dependent vascular homeostasis [64,65,66,67]. ROS mediate cellular effects of various stimuli and can modulate cell function via redox-sensitive signalling transduction, causing vasoconstriction, vascular smooth muscle cell proliferation, and the promotion of vascular inflammation [39,68].

Inflammation is closely associated with ROS and hence endothelial dysfunction. It, however, creates a ‘chicken or egg’ problem because endothelial dysfunction can in turn promote inflammation. In fact, without modulation or alteration of endothelial function in physiological or pathophysiological courses of inflammation, neither initiation nor propagation of the inflammatory process is conceivable. To initiate inflammation, endothelial cells have to be activated to ensure endothelial cell/leukocyte interaction by expressing adhesion proteins (e.g., P-selectin) and loosening endothelial cellular junctions to enhance tissue recruitment of inflammatory cells. This activation can either be rapid and transient or slow and more persistent. During the course of slower and more persistent activation, endothelial cells may produce pro-inflammatory cytokines (e.g., TNF-α, IL-1) which further recruit inflammatory cells. Endothelial cell activation and production of these cytokines enhances the expression of adhesion molecules (ICAM-1, VCAM-1). This creates a milieu for further activation of inflammatory cells and their recruitment, ultimately leading to persistent vessel wall inflammation [39,69,70,71].

#### 2.2.2. Interleukin 17 (IL-17)-Dependent Inflammation

**Figure 3:**
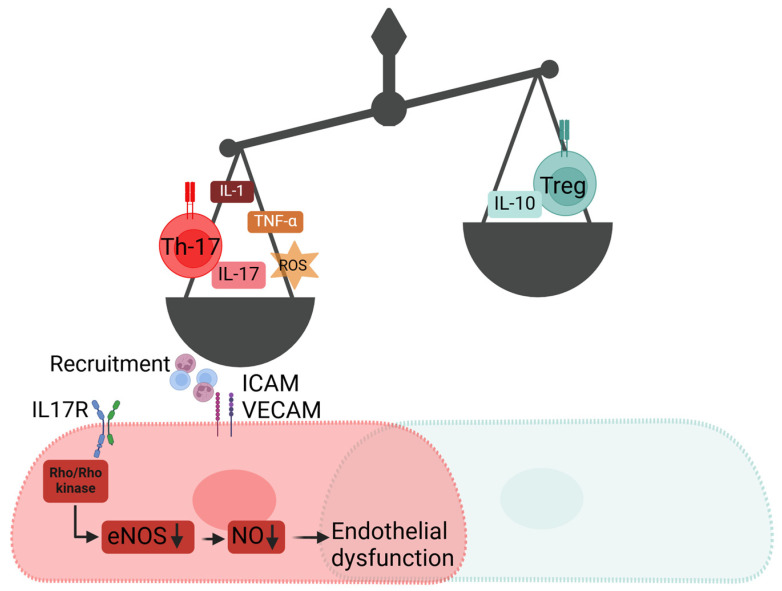
Schematic representation of inflammatory imbalance and effectors mediating inflammation-dependent endothelial dysfunction.

While it is undisputable that modulation of endothelial cell function is essential for initiation and progression of inflammation, inflammation in turn also affects endothelial cell function. In this regard, uncontrolled inflammation during hypertension may cause/aggravate endothelial dysfunction. While all branches of the immune system, from innate to adaptive immune cells, are involved in inflammation during hypertension [72,73,74], particular interleukins mediate the organ damage. Pro-inflammatory IL-17 is known to mediate inflammatory-target organ damage during hypertension. Ngyen et al. demonstrate that IL-17 causes endothelial dysfunction during experimental hypertension in mice [75]. IL-17 phosphorylates the inhibitory residue Thr495 of eNOS via activation of Rho-kinase (Figure 2 and Figure 3). As a result, IL-17 aggravates the rise of arterial blood pressure by decreasing NO bioavailability. Conversely, genetic knockout or elimination of IL-17 by antibody treatment blunts the progression of ANGII-induced hypertension and development of target organ damage, which is associated with maintained endothelial function and NO bioavailability [75,76]. Other approaches such as inhibition of IL-17 production and IL-17-dependent inflammation also protect from endothelial dysfunction, target organ damage, and progression of hypertension [77]. We showed that the inhibition of pro-inflammatory recruitment and the activation of anti-inflammatory Tregs suppress vessel infiltration, production of IL-17, and propagation of inflammation. These effects protect from endothelial dysfunction and target organ damage, accompanied with the rise in the systolic blood pressure. Dysregulation of the Th17/Treg axis is an important mechanism in other models of endothelial dysfunction. Mihalj et al. report the effects of a high-salt diet (HSD; 4% NaCl) changing the circulating immune cell landscape [78]. While HSD suppresses Tregs, it increases Th17 cell counts and by that reduces the Treg/Th17 ratio, which promotes inflammatory vascular damage. Therefore, the stabilisation of Tregs is an important counter effector in ANGII-induced hypertension organ damage as these cells produce anti-inflammatory IL-10 which may resolve inflammation [77]. IL12 is produced via several inflammatory cells, which is another anti-inflammatory interleukin implicated in pro-resolving actions during hypertension and protecting from ANGII-induced hypertensive vessel remodelling [79].

#### 2.2.3. Beneficial Effects of Modulation of Inflammation and Oxidative Stress on Endothelial Function

**Figure 4:**
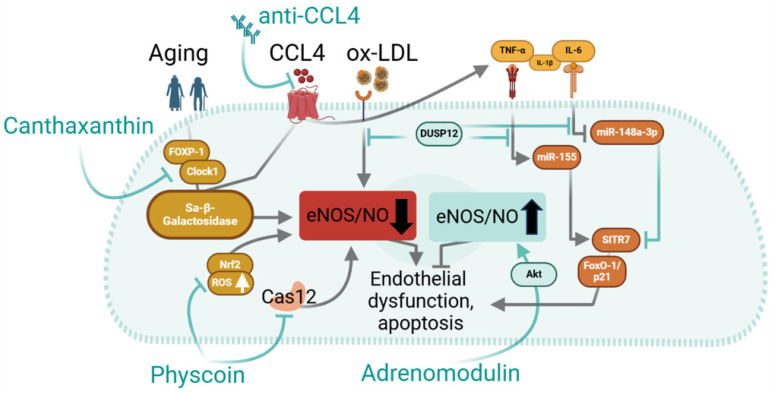
Possible experimental targets modulating inflammation and adverse effects of metabolic stress which may protect from endothelial dysfunction.

While the detrimental effects of various stimuli on endothelial function are mediated via activation of specific intracellular signalling pathways, the suppression of the mechanistic axes, which protect endothelial function, is essential for cellular dysfunction (Figure 4). Li et al. demonstrate a protective role of dual specificity phosphatase 12 (DUSP12) in oxidised low-density lipoprotein (ox-LDL)-induced dysfunction in human umbilical vein endothelial cells (HUVEC) [80]. By overexpressing DUSP12 in ox-LDL treated HUVECs, the authors demonstrate that the cells were protected from apoptosis. Under this condition, HUVECs also released less pro-inflammatory cytokines such as TNF-a, IL-1b, and IL-6 indicating amelioration of endothelial pro-inflammatory activation. Overexpression of DUSP12 preserved endothelial function by alleviating ox-LDL-induced decrease in eNOS/NO.

While all cells physiologically age, inflammation and oxidative stress force the ageing process, leading earlier to imminent cellular dysfunction. The concept of delaying ageing to preserve endothelial function and overall vascular health has long been in focus of experimental research. Recently, Wang et al. reported the protective role of canthaxanthin, a naturally occurring carotenoid, in isolated arterial and venous endothelial cells, as well as in ageing mice [81]. The authors could demonstrate that canthaxanthin decreases the accumulation of Sa-β-galactosidase (cell senescence marker) when applied in cell culture or in mice. Canthaxanthin also decreased the release of TNF-α, IL-1β, and IL-6 by endothelial cells under oxidative stress. Mechanistically, canthaxanthin may act via FOXP1 (Forkhead box P1)-dependent upregulation of Clock1, which in turn may act as a transcription factor regulating the cellular molecular circadian clock and stem cell senescence [82], indicating that endothelial and stem cells may share a Clock1-dependent mechanism of cellular senescence. While Clock1 seems to delay cellular senescence, chemokine C-C motif ligand 4 (CCL4) has been shown to promote it. Chang et al. demonstrate that CCL4 is upregulated in arterial endothelial cells, as well as endothelial progenitor cells of elderly subjects [83]. Upregulation of CCL4 in these cultured endothelial cells leads to increased production of reactive oxygen species and pro-inflammatory cytokines (TNF-α, IL-1β, IL-6) along with a decrease in Sa-β-galactosidase. Inhibition of CCL4 in vitro or use of neutralising antibodies in mice, improved endothelial function in this experimental model.

While endothelial cells release pro-inflammatory cytokines during their activation, when applied, these cytokines have detrimental effects on endothelial function. IL-6 has been shown to induce endothelial dysfunction and apoptosis largely by mediating oxidative stress and mitochondrial injury [84]. IL-6 induces endothelial release of further pro-inflammatory cytokines such as TNF-α and IL-1β, thereby aggravating a pro-inflammatory activation of endothelial cells. IL-6, on the one hand, acts via increased levels of sirtuin 7 (SIRT7) [84], which is a known promoter of apoptosis [85], and on the other hand, by decreasing miR-148a-3p, a micro RNA opposing effects of SITR7. This relationship of IL-6 and miR-148a-3p/SITR7 has been tested in human arterial endothelial cells, where upregulation of miR-148a-3p decreased IL-6/SITR7-mediated mitochondrial damage and apoptosis [84]. Some miRNAs, however, mediate more detrimental effects in endothelial cell. TNF-α upregulates miR-155, which in turn leads to SIRT1-dependent endothelial cell apoptosis in a FoxO-1/p21pathway [86].

In search of protective agents to prevent endothelial dysfunction, a chemical ingredient of rhubarb—physcion—was tested in mice challenged by a high fat diet [87]. The application of physcion in mice with HFD showed better endothelium-dependent relaxation associated with a low rate of apoptosis and low endothelial oxidative stress. Improved endothelial function seems to depend on activation of eNOS/Nrf2 (nuclear factor-E2-related factor) signalling and stabilisation of NO production, whereas low apoptosis probably resulted from inhibition of caspase-12 signalling. Nrf2 has also been implicated in ROCK 2 (Rho-kinase 2) dependent endothelial dysfunction in another model of hypertension (ANGII-infusion) [88]. The mechanistic axis made up by Akt/eNOS is well known to regulate NO production and by that maintaining homeostatic functions of endothelial cells. The high fat diet challenges this pathway leading to decreased NO production and ultimately to endothelial dysfunction. This process has a long time dynamic, which partly rests on the resistance of the endothelial cells toward a dysfunctional shift mediated via protective cellular mechanisms. Adrenomodulin (ADM), a multifunctional peptide, is elevated in obese individuals. ADM acts via its receptors and stabilises the Akt/eNOS axis to maintain NO production. A protective role of ADM is supported by a study on HFD treated mice which showed that application of ADM improved endothelium-dependent vessel relaxation and blunted the rise in arterial blood pressure [89].

### 2.3. Metabolic Disorders

**Figure 5:**
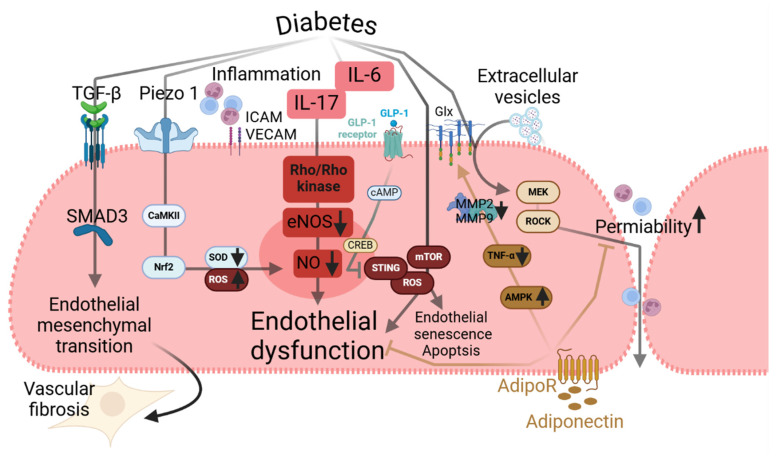
Cellular mechanisms mediating endothelial dysfunction during metabolic stress.

Along with hypertension, metabolic diseases such as type 2 diabetes are associated with endothelial dysfunction. Although not all patients with hypertension are obese or have type 2 diabetes, many patients have both hypertension and metabolic disorders, which aggravates endothelial dysfunction (Figure 5). In a limited observational study Koppara et al. assessed endothelial function and atherosclerotic vessel damage in patients with chronic kidney disease (CKD) with and without type 2 diabetes [90]. The patients with type diabetes had worsened endothelial function with decreased NO and increased IL-6 levels along with more atherosclerotic plaques compared to non-diabetic CKD patients. This study indicates that the diabetic patients with CKD have profoundly increased risks of cardiovascular complication compared to CKD patients without type 2 diabetes. Endothelial dysfunction may even precede development of diabetes. Li et al. assessed triglyceride-glucose (TyG) index and flow mediated dilatation in over 1000 patients without diabetes mellitus [91]. TyG is calculated by fasting triglyceride (TG) (mg/dL) × fasting plasma glucose (FPG) (mg/dL)/2, which is considered a surrogate marker for insulin resistance. They found that patients with increased TyG index had lower flow-mediated vasodilatation, possibly due to endothelial dysfunction. This association was stronger in patients under 60 years old without diabetes mellitus. Zaib et al. assessed markers of endothelial dysfunction in type 2 diabetic and non-diabetic patients and showed that serum IL-1, ICAM, and VECAM were profoundly higher in patients with type 2 diabetes [92]. It is claimed that these markers help to assess the prognosis of developing peripheral artery disease in type 2 diabetic patients. In fact, anti-diabetic medications such as glucagon-like peptide-1 receptor agonists (GLP-1 RAs) improve endothelial function during diabetes. It is well accepted that GLP-1 RAs reduced cardiovascular and renal complications during diabetes. Recently, He et al. used single cell RNA sequencing and immune staining to demonstrate that these receptors are downregulated on retinal endothelial cells in diabetic patients. This effect may critically contribute to diabetic retinopathy [93]. Treatment with GLP-1 RAs restores GLP-1R expression in endothelial cells and slows the progression of diabetic retinopathy. GLP-1 RAs correct endothelial cell function by improving mitochondrial function. Improvement of mitochondrial and endothelial function were associated with activation of cAMP-response element binding protein (CREB) and consequent inhibition of STING signalling, which is involved in autophagy, senescence, and apoptosis.

Dysfunctional endothelium may affect various physiological processes. Vuong et al. reported that endothelial dysfunction associated with type 2 diabetes may cause impaired wound healing [94]. By comparing endothelial cells from healthy and type 2 diabetic donors, the authors demonstrate that the endothelial cells of healthy donors showed better cell survival and antigenic abilities compared to those from donors with type 2 diabetes. The cells of donors with type 2 diabetes were more prone to TGF-β-Smad3-dependent endothelial mesenchymal transition, an indicator of endothelial dysfunction and phenotypic alteration [58,59,60], than the cells of healthy nondiabetic donors. Type 2 diabetes is associated with high cerebrovascular permeability, which is considered to be one factor involved in genesis of cognitive impairment. Gustafson et al. found that extracellular vesicles collected from plasma of a diabetic mouse model (Leprdb/db) induced rapid disruption of the endothelial barrier upon injection in non-diabetic mice [95]. This increased endothelial permeability depended on the endothelial MEK/ROCK pathway. However, in this study no inflammatory recruitment was observed, most likely due to the short duration of the experiment. Zhang et al. reported that mechanosensitive signalling is one of the key mechanisms in high glucose induced endothelial dysfunction [96]. Endothelial specific conditional Piezo1 knockout mice were protected from endothelial dysfunction and damage under hyperglycaemic condition. More eNOS and less inflammatory cells were observed in the thoracic aorta with less adverse remodelling. Using HUVECs, the authors reported that Piezo1 acts via the Ca^2+^-induced CaMKII-Nrf2 axis and reduces the activity of superoxide dismutase, which in turn aggravates oxidative stress in endothelial cells. Islam et al. demonstrate that improvement of endothelial function was associated with protection from metabolic dysfunction [97]. In a mouse model with tamoxifen-induced knockout of mammalian target of rapamycin (mTOR), which accelerates endothelial senescence and vascular dysfunction [98], there was a decrease in senescence markers and less oxidative stress and vascular inflammation. Interestingly, knocking out endothelial mTOR and protection from endothelial dysfunction improved glucose tolerance in mice, which was associated with reduced hepatic gluconeogenesis and better lipid tolerance. More importantly, these effects were not dependent on pancreatic beta cell function or improved insulin sensitivity, which emphasises the role of endothelial cells on glucose transport or metabolism aside from insulin stimulation. Fawaz et al. demonstrates the protective role of adiponectin on glomerular endothelial glycocalyx in a mouse model of diabetic kidney diseases (DKD) [99]. Mice treated with adiponectin were protected from albuminuria by attenuating shedding of glycocalyx. Adiponectin acts via its receptor and activates AMPK reducing TNF-α release from glomerular endothelial cells, which in turn inhibits the expression of matrix metalloproteinase (MMP2 and MMP9), which are associated with glycocalyx shedding.

## 3. Endothelial Mechanisms of Vascular Remodelling

### 3.1. Endothelial AT1 Receptors

ANGII mediates its effects via pharmacologically and functionally identical AT1_A_R and AT1_B_R. Hence, adverse effects of ANGII in cardiovascular tissues (e.g., vessel and heart) are likely mediated via direct actions on AT1R located on vascular smooth muscle cells or cardiomyocytes. However, Sparks et al. completely deleted AT1_A_R on large artery smooth muscle cells, with a preservation of 50% expression of AT1_A_R on the smooth muscle cells of the small resistance vessels [100]. The authors demonstrate that mice lacking AT1_A_R on vascular smooth muscle cells were not protected from ANGII-induced medial hypertrophy. Interestingly, persistent medial hypertrophy was seen despite the fact that the mice lacking AT1_A_R exhibited less vascular oxidative stress. Recently, it has been demonstrated that deletion of AT1_A_R on cardiomyocytes does not protect from ANGII-induced cardiac hypertrophy [101]. In mice lacking the AT1_A_R on cardiomyocytes, ANGII infusion over 4 weeks resulted in the same extent of cardiac hypertrophy and fibrosis as in control WT mice challenged with the same ANGII protocol. In both studies, the authors concluded that AT1_A_Rs on smooth muscle cells or cardiac myocytes are not required in ANGII-induced cardio-vascular remodelling. On the one hand, these studies point to possible mechanisms of ANGII-induced remodelling without directly employing its receptors on target cells like vascular smooth muscle cells or cardiomyocytes. This would strengthen a potential role of other regulatory systems, such as inflammation, along with paracrine actions of dysfunctional endothelial cells in the mediation of hypertension-induced organ remodelling. On the other hand, it is worth noting that in these studies the authors deleted only the AT1_A_R without affecting the expression of AT1_B_R. Therefore, one might interpret those findings assuming that the deletion of only AT1_A_R on smooth muscle cells or cardiomyocytes is not sufficient enough to achieve protection from ANGII-induced cardio-vascular remodelling. Conversely, these studies may further strengthen the obligatory role of a dysfunctional endothelium in the progression of target organ damage, and most importantly, vascular remodelling during hypertension. In this regard, Rateri et al. demonstrated that deletion of AT1_A_ receptors on endothelial cells protected LDL knockout mice from the ANGII-induced development of ascending aortic aneurisms [33]. Interestingly, deletion of AT1_A_R on smooth muscle cells or bone marrow derived-cells did not protect these mice. The findings point to the specific role of endothelial AT1_A_Rs in the development of ANGII-induced ascending aortic aneurism. In a later study, the authors demonstrated that the same mouse strain lacking endothelial AT1_A_R was not protected from abdominal aortic aneurism or atherosclerosis [102], suggesting potentially different roles of the endothelium in various regions of aorta. Altogether, the studies described indicate that ANGII induced arterial remodelling may critically depend on activation of the endothelium via its AT1Rs and may include activation of AT1_A_R and AT1_B_R.

### 3.2. ET-1—A Mediator of Endothelial Crosstalk with Vascular Smooth Muscle Cells and Its Role in Arterial Remodelling

Although the term endothelial dysfunction entails a number of alterations in endothelial function, the decrease in NO production/bioavailability and consequent overall loss of endothelial protective effects and maintenance of vascular homeostasis is considered a prime hallmark of endothelial dysfunction [46]. A most instantaneous result of decreased endothelial NO production is the increase in vessel tone, which may affect arterial pressure. Indeed, experimental studies demonstrate that infusion of L-NMMA, a pharmacological eNOS inhibitor, causes immediate elevation of arterial pressure [103,104]. Likewise, mice with a genetic deletion of the endothelial eNOS gene exhibit increased arterial blood pressure [105]. NO has an important autocrine inhibitory effect on ET-1 synthesis, whereas ET-1 in turn inhibits eNOS activity (Figure 2) [106]. Thus, declined NO production may increase ET-1 release from endothelial cells further increasing vascular tone and arterial blood pressure. ET-1 acts via ET_A_ and ET_B_ receptors. Selective ET_A_ blockade lowers systolic blood pressure in hypertensive rats [107]. In patients with primary hypertension, both selective and combined inhibition of ET-1 receptors lowers arterial pressure [108]. Thus, rather a dysbalance of NO and ET-1 production may explain the increases in vessel tone and arterial pressure. Moreover, both the decrease in NO and the increase in ET-1 release contribute to arterial stiffening, which is important in the vessel remodelling process and development of sustained hypertension. Wilkinson et al. demonstrate that the infusion of L-NMMA increases arterial stiffness assessed by increased pulse wave velocity, whereas supplementation of a NO donor decreases arterial stiffness [109]. Moreover, a single or combined blockade of ET-1 receptors decreases arterial stiffness [110].

We have recently proposed a novel mechanistic axis entailing the endothelial AT1R—ET-1/ET_A_ receptor axis for activation of latent pro-matrix metalloproteinase 2 (pro-MMP 2) in the aortic wall (Figure 2) [111,112]. MMP-2 is a gelatinase which degrades elastin and promotes fibrosis in the arterial wall, causing a major dysbalance between elastic and non-elastic components [113]. By degrading elastic fibres (combined with fragmentation) and forcing collagen deposition it causes arterial stiffening. MMP2 is expressed both in vascular endothelial and smooth muscle cells. Interestingly, mechanisms regulating this expression are cell specific. In endothelial cells, ANGII employs AT1R and activates the JNK/c-jun/AP-1 pathway leading to increased latent pro-MMP2 expression [112]. In vascular smooth muscle cells, ANGII-induced activation of AT1R/JAK2/STAT3 leads to augmented pro-MMP2 expression. Pro-MMP2, unlike other MMPs, is hardly activated by proteases but requires a complex interaction of multiple factors to ensure its activation. In the vessel wall, upon release as a latent pro-MMP2 into the extracellular matrix, pro-MMP2 is catalytically cleaved (activated) by membrane type MMP 1 (MT1-MMP) expressed on the vascular smooth muscle cells. We demonstrated that this key process, which can limit the biological efficacy of MMP 1 is regulated by the endothelial cell [111,112]. Activation of pro-MMP2 by MT1-MMP is the last step of a precisely regulated activation cascade. ET-1 released by the endothelium activates furin in smooth muscle cells in a paracrine manner via acting on ET_A_ receptors. Furin cleaves MT1-MMP and αvβ3 integrin from their precursors, which then translocate to the cell membrane. αvβ3 integrin binds latent pro-MMP2 and the MT1MMP catalytically cleaves it to active MMP2 on the smooth muscle cell membrane. ANGII activates this pathway and causes the expression of pro-MMP2 in endothelial and smooth muscle cells to increase via two distinct pathways (see above). It also increases αvβ3 integrin and MT1-MMP expression in smooth muscle cells without affecting furin activity. However, when furin activity is increased via ET-1 released from the endothelium, this not only assures a homeostatic regulation of pro-MMP2 activity in the arterial wall under physiological conditions but, under conditions of endothelial dysfunction, stimulates, in the presence of ANGII, the activation of latent pro-MMP2 via the ET-1/ET_A_/Furin pathway.

## 4. Sex-Specific Differences in Endothelial Function and Dysfunction

### 4.1. Sex-Specific Differences in Endothelium-Dependent Vessel Tone Regulation

**Figure 6:**
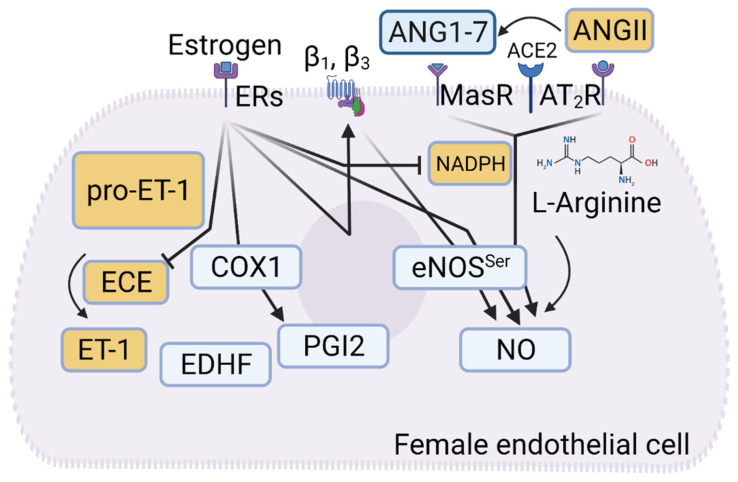
Oestrogen acts via its receptors and maintains a balanced production of vasoactive peptides in favour of relaxing factors. Oestrogen acts partly directly but also via the expression of β_1_- and β_3_-adrenoceptors, which in turn activate eNOS (shown in rat model). The expression of more ACE2 on female endothelial cells allows it to effectively metabolise ANGII to ANG 1-7 and activate eNOS via MasR and AT2R, which are also expressed in female endothelial cells.

Oestrogen has a major impact on vessel tone which is attributed mainly to increased endothelial NO bioavailability via stimulation of the endothelial PI3K/Akt/eNOS mechanistic axis (Figure 6) [114,115]. This increased NO production may also be linked to the antioxidant effect of oestrogen in endothelial cells by inhibiting NADPH oxidase expression and decreasing the production of ROS [116]. Oestrogen also increases the production of vasodilatory PGI2 by increasing the expression of COX-1 in endothelial cells [117]. This COX-generated PGI2 acts differently in aged male spontaneously hypertensive rats (SHR), where it causes vasoconstriction because of a preferred action on thromboxane A2 receptors instead of PGI2 receptors. This, in turn, may contribute to the development of endothelial dysfunction in male SHR [118]. The importance of the thromboxane receptor in this sex-specific vasoconstriction was confirmed by alteration in acetylcholine-induced vasorelaxation in males but not females upon blocking of thromboxane receptor [119].

Sex related differences in EDHF mediated relaxation have been uncovered in the “EDHF mouse”. These mice lack eNOS and COX and develop hypertension. However, female mice are resistant to hypertension owing to the compensatory effect of EDHF [120]. In young female rats, the EDHF-mediated relaxation of resistance vessels is stronger compared to postmenopausal rats. This is explained by enhanced EDRFs mediated vessel relaxation due to oestrogen action, associated with increases in basal and acetylcholine-stimulated Ca^2+^ transients in endothelial cells [121]. A role of EDHFs for sex differences in vessel relaxation was also reported for porcine coronary arteries. Female vessels relax more strongly than vessels of males [122]. It also appears that there are sex-specific differences in the mechanisms of EDHFs mediated relaxation. While EDHFs caused relaxation in female coronary arteries via activation of two distinct calcium activated potassium channels, SK_Ca_ and IK_Ca_, in male vessels only the SK_Ca_ was objectified.

Sex-differences are also demonstrated for the responses of male and female blood vessels toward adrenergic stimuli. Forearm blood flow in men and women responds differently toward norepinephrine infusion, which causes a stronger decrease in flow in men compared to women. Conversely, selective β-adrenergic stimulation increases forearm blood flow more profoundly in women, compared to men [123,124]. An important mechanism of the sex-specific differences toward adrenergic stimuli may result from sex differences in endothelial β-adrenoceptors. As previously reported, endothelial cells in female rat aorta express more endothelial β_1_- and β_3_-adrenoceptors compared to age matched male rats [9,10]. We also demonstrated that these sex-specific expressions of endothelial β_1_- and β_3_-adrenoceptors are oestrogen dependent, whereas other sex hormones do not affect the expression of these adrenoceptors on endothelial cells (Figure 6). The different expression pattern has a major impact on adrenergic vessel tone regulation in both small resistance vessels as well as large arteries. Aorta and mesenteric arteries respond with less vasoconstriction to norepinephrine stimulation, which is explained by dual α- and β-adrenoceptor stimulation. Because female endothelial cells express more β_1_- and β_3_-adrenoceptors, upon adrenergic stimulation, their NO-dependent relaxation is enhanced, counteracting the α-constrictive effect or augmenting the β-dilatory effect on smooth muscle. Of note, the expression of β_1_- and β_3_-adrenoceptors is also higher in the human mammary arteries of women compared to the men in the same age group. This is accompanied with a sex-related difference in the adrenergic vessel response, where vessels of women constrict less to norepinephrine but relax more to isoproterenol or selective β_3_-adrenoceptor stimulations [9,10].

Besides female sex hormones, androgens also exert effects on endothelial cell function. Ruamyod et al. report that Ca^2+^-gated potassium channels mediate the endothelium-dependent vasodilatory effect of testosterone. Androgen via its G-protein coupled receptors and PKA activates these potassium channels leading to endothelial cell hyperpolarisation and release of endothelial vasodilatory factors (NO, PGE) [125]. In a limited clinical study in patients with androgen excess polycystic ovary syndrome, Usselman et al. propose that androgen may drive endothelial dysfunction and aggravate cardiovascular risks typically related to the disease [126]. While androgen in this disease model may have detrimental effects, oestrogen may play a protective role in preserving endothelial function. Mechanistically, the authors propose that androgens act via supressing NO production in this disease model. Androgens also seem to have effects on endothelial progenitor cells. Lam et al. show that there was a correlation between serum testosterone and the levels of endothelial cell progenitors in patients with coronary artery disease after catheterisation [126]. Based on a mouse model of myocardial infarction, the authors propose that testosterone may promote neovascularisation by enhancing endothelial progenitor cells.

### 4.2. Sex-Specific Differences in Development of Endothelial Dysfunction and Arterial Remodelling

Based on the above-described sex-specific differences in the production of endothelial vasoactive factors and their effects on vessel tone, it is conceivable that these differences may affect the homeostatic role of endothelium with respect to physiological vessel function and pathological vascular dysfunction. Endothelial dysfunction is observed in the model of SHR [127,128]. These rats show sex-differences in development of hypertension associated with differences in cardiovascular remodelling, which are largely attributed to sex-differences in ACE2, AT_2_R, and MasR, all of which are found to be stronger expressed in females than in males [128,129]. ACE2 converts ANGII to ANG 1–7, which in turn acts on MasR. This mechanistic axis activates eNOS and triggers NO production. Importantly, AT_2_R have opposing effects that counteract those of AT_1_R stimulation. Along with MasR, ANGII/AT_2_R also activates eNOS and thereby supports NO production. We advanced this knowledge by showing that in the rat SHR model, endothelial dysfunction develops along with two other parameters, namely arterial remodelling and activation of latent pro-MMP2 [128]. During ageing the endothelium-dependent relaxation declines first in male SHRs at an age of 14 weeks. Along with endothelial dysfunction, arterial remodelling occurs with aortic fibrosis and degradation of elastin first in male SHRs. Further, the activation, but not expression, of latent pro-MMP2 strongly correlates with the progression of endothelial dysfunction (r^2^ = 0.87) [128]. The endothelium controls activation of latent pro-MMP2 in physiological conditions via ET-1 [111,112], and hence stimulated ET-1 production activates latent pro-MMP2 under conditions of endothelial dysfunction. This likely contributes to vessel remodelling, the onset of which is considerably delayed in female SHRs, most likely because of longer preservation of endothelial function [128].

## 5. Evaluation of Endothelial Dysfunction and Its Therapeutic Implications

While endothelial cells may lose their protective function and transform into dysfunctional cells, we lack a clear characterisation of the steps mediating this transition. However, endothelial type I and type II activations are considered as initial events on the way leading to endothelial dysfunction [130,131,132]. Type I activation is rapid and does not necessarily include irreversible morphological or functional changes. Type II activation on the other hand involves a phenotype change in endothelial cells, turning more into a pro-inflammatory phenotype. Along this way of transition, several markers, tested via laboratory or clinical methods, could be helpful to assess endothelial function and cardiovascular risk. A balanced production of a number of factors by the healthy endothelium, such as NO, von Willebrand factor, tissue factor, number of adhesion molecules or chemokines, can be assessed with routine laboratory methods. Furthermore, components of the glycocalyx, such as heparan sulphate and syndecan-1, were shown to reflect endothelial function [133,134]. Lastly, there are circulating endothelial and their progenitor cells, assessment of which may give insights into the capacity of vascular repair upon endothelial injury [135].

Besides the assessment of markers using laboratory methods, the evaluation of the endothelial function by the assessment of flow mediated dilatation has long been used [136]. This non-invasive and cost-effective procedure has permitted the assessment of the endothelium-dependent NO mediated vessel relaxation by triggering effects of increased shear stress on endothelial NO production. Recently, Holder et al. reported age- and sex-specific reference values of flow mediated dilatation. While the authors clearly demonstrate a general age dependent decline in flow mediated dilatation in healthy individuals, men showed a negative, curvilinear relation between flow mediated dilatation and age, whereas women revealed a negative linear relation with fast decline [137]. Several other clinical methods have been employed to test endothelial function, such as venous occlusion plethysmography, laser Doppler flowmetry, and peripheral artery tonometry. The latter allows the automated assessment of microvascular endothelial function [138,139,140].

Irrespective of whether endothelial/vascular dysfunction may be a cause or consequence of hypertension, both the increase in arterial pressure and endothelial dysfunction are important targets of therapy. While lowering/normalising arterial pressure is a major target of European and American guidelines, there are no specific recommendations dealing with the improvement of endothelial function. It seems that this is rather appreciated as a beneficial side effect of antihypertensive or cardiovascular therapy. Nevertheless, widely used inhibitors of ACE and angiotensin II type 1 receptor (ARBs) largely improve flow mediated dilatation in patients [141,142,143,144]. Lowering ANGII levels may improve endothelial function via direct and indirect effects. Inhibition of adverse effects mediated by the ANGII/AT1R axis, may stabilise eNOS activity and improve NO production. In turn, lowering the stimulatory effect of ANGII/AT1R axis on ET-1 production [111], the balance may be tilted in favour of more NO and less ET-1. This balance may also be supported by accumulation of bradykinin and stimulation of NO production via bradykinin receptors 1 and 2 [145]. While the latter has an indirect effect on endothelial function, there are more direct beneficial effects of inhibition of ANGII, especially by lowering its pro-inflammatory actions, reducing oxidative stress, improving DDAH1 activity and lowering ADMA levels [145].

Exercise is one of the most effective non-pharmacological interventions to counter the risk factors for development of hypertension and cardiovascular diseases [146,147,148,149,150]. Beneficial effects of exercise on endothelial function were shown as early as 1986 by Sinoway et al. demonstrating improved forearm blood flow in tennis players [147]. The impact of endothelial function has also been tested in exercise based cardiac rehabilitation studies in patients with coronary artery disease, myocardial infarction, and heart failure [151,152,153,154]. By testing flow mediated dilatation, improved endothelial function after exercise-based rehabilitation has also been reported in patients with COPD [155]. Finally, an increased number of circulating endothelial progenitor cells after exercise-based rehabilitation has been demonstrated, indicating the role of exercise in endothelial regeneration [151,152]. Various mechanisms including reduced oxidative stress due to increased activity of superoxide dismutase and downregulation of NOX, along with increased eNOS phosphorylation and mobilisation of endothelial progenitor cells, have been proposed [134].

## 6. Conclusions

Endothelial dysfunction not only results in the loss of its homeostatic functions, but dysfunctional endothelium may critically augment vascular damage by mediating adverse vessel remodelling. A decrease in the bioavailability of NO and an enhanced ET-1 production have important consequences such as vascular inflammation and enhanced activation of MMP2. Based on this, endothelial dysfunction may be considered a pathophysiological factor in vessel remodelling and in overall cardiovascular disease development during hypertension. Hence, besides the management of arterial blood pressure, a better understanding of pathophysiological mechanisms is critical in the development of preventive and therapeutic strategies of hypertension and related organ damage. Although mechanisms of endothelial dysfunction are multifactorial, the role of RAS and ANGII in the mediation of endothelial dysfunction is central. While ANGII exerts direct adverse effects on endothelial function, additional effects on the immune system and on metabolic pathways lead to a complex system of interactions eventually resulting in endothelial dysfunction. Since ANGII exerts its functions via different cell surface receptors, the potentially redundant roles of endothelial AT_1a_R and AT_1b_R need to be better characterised, along with their downstream signalling pathways. Furthermore, ACE2, MasR, and AT_2_R exhibit higher expressions in female as compared to male tissues. Still, these proteins are expressed on male endothelial cells with no rudimental functional roles as objectified to date. In addition, it is still unknown whether endothelial ACE2 and MasR contribute to the maintenance of homeostatic functions of the endothelium and whether the differences in expressions of ACE2 and MasR play a causative role in the development of endothelial dysfunction, and hence contribute to overall vessel damage during hypertension.
